# Hospital Housekeeping

**Published:** 1898-08-27

**Authors:** 


### HOSPITAL HOUSEKEEPING.

By a late Matron.

II.—Marketing.

In several American hospitals of which I have had experience it was one of the duties of the hospital housekeeper to go "down town to market" every morning, and there to choose extras, and to buy fruit, fowls, fish, &c., just according to whether these happened to be cheap and plentiful.

There are many arguments in favour of such a system, for the staffs and patients were better fed than any other hospital inmates I have ever met with—save, perhaps, in Germany, that land of clever housekeeping. A good manager who follows the market day by day soon learns to get excellent value for her money, and is able to form a fairly shrewd idea of the extent to which prices are likely to keep up. If a barrel of apples is shortly to be needed experience teaches her to recognise the signs of the market, and she aptly diagnoses the exact moment when she is likely to get the best apples for the least price. Tomatoes, celery, and even asparagus, to say nothing of strawberries and other tempting fruit, are often astonishingly low in price—in the market, not in the shops—and where some of the housekeeping is done from day to day the hospital patients and staffs get the benefit of a glut in the fruit and extra delicacy market. It cannot be denied by anybody who has experienced this variety of housekeeping that it has much to recommend it. The rigid system which arranges the diets three months ahead, as it were, and decrees by the unalterable law of contract that the hospital inmates shall on specified days during the quarter consume a certain portion of mutton and rice or beef and tapioca, the *régime* going on like the brook—for ever in an unchanging monotony—has many drawbacks. I remember now with a nauseating exactness how every winter Sunday in my old training school we used to have black currant jam tart. The hatefulness of its monotony caused us to regard black currant jam as one of the most refined tortures of hospital life. And the system was so senseless. Other kinds of jam would have been equally cheap, and we would not have minded if the other jam had been equally horrid, so long as it was a different kind of horrid! The alternative—how well I remember it—was the invariable rice or tapioca. Now, if our housekeeper had only been to market the day before, what infinitely nice Sunday dinners we might have had!

During a lengthened experience of American hospitals, I never remember the nurses knowing what dinner to expect just because the calendar said Sunday or Monday. Their food depended—as it should—on the state of the market and the plentifulness and cheapness of certain supplies. To feed an institution on "a plan" which leaves out all considerations of "unexpectedness" and allows no variety is to produce a lower standard of health than is attained where change and surprises are introduced into the dietary.

There is no question that our present system of hospital housekeeping leads to nurses spending a great deal more than they can afford in diet and extras, such as cocoa, jams, &c. Their diet is so monotonous and "expected" that it induces a loss of appetite, which causes them to supplement their "home" diet by chocolates, pastry, and other such "good things" of life.

The same arguments apply to the patients in our hospitals. To take one article of diet which, above all others, is beloved of the Londoner—fish—how rarely this is served to the sick. It is ordered as a luxurious "extra"—a proceeding which, from the housewifely point of view, is absurd, for fish bought in large quantities at the proper place and in the right season is extremely cheap. There is no reason why it should be doled out in institutions as though it were worth its weight in gold, and I have never been able to understand why the patient in hospital "on fish" should, day after day, week after week, get the same fish—usually boiled cod.

There are many fish in the sea, and there are many kinds of fish equally as cheap as cod, which, as a matter of fact, is not at all typically economical, so that the sick person need not have that same inevitable plate of cod brought to his bedside with wearisome persistence. I have said my say before in The Hospital pages as to the rice pudding. I have never been able to explain—nor has anybody whom I have questioned on the subject been able to throw any light on the reason—why the old tradition survives that rice, and rice alone, is the one pudding whereon the sick may be restored to convalescence. Nurses who have grown grey in ward service, matrons of much experience, have never yet given a satisfactory explanation as to why, of the many hundreds of light, inexpensive, and nourishing puddings, rice and custard hold the hospital dietetic field. I might remark, in passing, that custard is a very expensive dish. It is an admirable compound of milk and eggs, but milk and eggs can be made up into so many delightful dishes that it is difficult to understand why baked custard should enjoy its present gigantic and widespread monopoly. The fact is, this matter of hospital housekeeping needs to be taken up in practical, definite spirit, and dealt with on a technical system.

There are many organised housewifery courses at present in vogue, and many well-trained housewifery and practical cookery teachers, so that it would be easy and inexpensive to introduce a course of such invaluable teaching into the training-school curriculum. At the same time, I am assured from much past experience that it would be quite useless to make an innovation of a course of hospital housekeeping unless at the same time a diet-kitchen were set up, in which the nurse should learn the practical preparation of every dish likely to be needed in the sick-room. And I would entirely deprecate the teaching of hospital housekeeping by means of theory, lecture, or text-book. To be of value, it must be learnt in store-room and market. The nurse-learner must study the raw material in th market, purchase it there, and dispose of it in the storeroom with due regard to the best way of keeping each individual article under the best conditions for preservation; and she must learn to turn the raw material into dainty dishes, and become proficient in the domestic bookkeeping essential to hospital housewifery.

I am fully aware that tentative steps have been made and are being made towards this teaching of hospital housekeeping which I now advocate, but I look forward to its universal adoption, and wait for the coming of a pioneer matron who will rise up and bring the system to a practical and thorough perfection, and establish the fact that the more housewifely the woman the better the nurse.

